# Usage Pattern Differences and Similarities of Mobile Electronic Medical Records Among Health Care Providers

**DOI:** 10.2196/mhealth.8855

**Published:** 2017-12-13

**Authors:** Yura Lee, Yu Rang Park, Junetae Kim, Jeong Hoon Kim, Woo Sung Kim, Jae-Ho Lee

**Affiliations:** ^1^ Department of Biomedical Informatics Asan Medical Center University of Ulsan College of Medicine Seoul Republic Of Korea; ^2^ Clinical Research Center Asan Medical Center Seoul Republic Of Korea; ^3^ Department of Convergence Medicine Asan Medical Center University of Ulsan College of Medicine Seoul Republic Of Korea; ^4^ School of Management Engineering Korea Advanced Institute of Science and Technology Seoul Republic Of Korea; ^5^ Medical Information Office Asan Medical Center Seoul Republic Of Korea; ^6^ Department of Pulmonary & Critical Care Medicine Asan Medical Center University of Ulsan College of Medicine Seoul Republic Of Korea; ^7^ Department of Emergency Medicine Asan Medical Center University of Ulsan College of Medicine Seoul Republic Of Korea

**Keywords:** mobile applications, electronic health records, physicians, nurses, communication, rounds, patient handoff

## Abstract

**Background:**

Recently, many hospitals have introduced mobile electronic medical records (mEMRs). Although numerous studies have been published on the usability or usage patterns of mEMRs through user surveys, investigations based on the real data usage are lacking.

**Objective:**

Asan Medical Center, a tertiary hospital in Seoul, Korea, implemented an mEMR program in 2010. On the basis of the mEMR usage log data collected over a period of 4.5 years, we aimed to identify a usage pattern and trends in accordance with user occupation and to disseminate the factors that make the mEMR more effective and efficient.

**Methods:**

The mEMR log data were collected from March 2012 to August 2016. Descriptive analyses were completed according to user occupation, access time, services, and wireless network type. Specifically, analyses targeted were as follows: (1) the status of the mEMR usage and distribution of users, (2) trends in the number of users and usage amount, (3) 24-hour usage patterns, and (4) trends in service usage based on user occupations. Linear regressions were performed to model the relationship between the time, access frequency, and the number of users. The differences between the user occupations were examined using Student t tests for categorical variables.

**Results:**

Approximately two-thirds of the doctors and nurses used the mEMR. The number of logs studied was 7,144,459. Among 3859 users, 2333 (60.46%) users were nurses and 1102 (28.56%) users were doctors. On average, the mEMR was used 1044 times by 438 users per day. The number of users and amount of access logs have significantly increased since 2012 (P<.001). Nurses used the mEMR 3 times more often than doctors. The use of mEMR by nurses increased by an annual average of 51.5%, but use by doctors decreased by an annual average of 7.7%. For doctors, the peak usage periods were observed during 08:00 to 09:00 and 17:00 to 18:00, which were coincident with the beginning of ward rounds. Conversely, the peak usage periods for the nurses were observed during 05:00 to 06:00, 12:00 to 13:00, and 20:00 to 21:00, which effectively occurred 1 or 2 hours before handover. In more than 80% of all cases, the mEMR was accessed via a nonhospital wireless network.

**Conclusions:**

The usage patterns of the mEMR differed between doctors and nurses according to their different workflows. In both occupations, mEMR was highly used when personal computer access was limited and the need for patient information was high, such as during ward rounds or handover periods.

## Introduction

For health care providers, mobile phones are emerging as clinical tools comparable in value to the stethoscope [1]. They are handheld tools that can be transported in a pocket, carried anywhere, and helpful in collecting valuable patient information [2,3]. However, smartphones are tools that are more ubiquitous than a stethoscope. Moreover, they can be linked to the hospital information system to identify patient information, deliver clinical knowledge, and assist in clinical decision making [4-7]. Smartphones are expected to play a role as potential medical devices beyond their conventional use for communication between health care providers [7-9]. Mobile health, manifested in part by smartphone use, is changing the paradigm of medical care with its mobility, compatibility with other devices, and powerful computing capability [4-7,10-13]. Health care providers, as well as patients, are beneficiaries of mobile health through various devices and apps [14-17].

Among the various applications for health care providers, mobile electronic medical records (mEMRs) are expected to be a solution to the lack of mobility of personal computer (PC)-based electronic medical records (EMRs) [18-22]. Correspondingly, mEMRs enable health care providers to freely exchange patient information and decision-making content irrespective of time and place in a secure environment [11,12,15,22,23]. According to recent reports on the acceptance of mEMRs or its effects, the users were found to be satisfied with the performance, efficiency of workflow, and improvement of communication [7,11,12].

Although previous studies on mEMRs have reported positive technological prospects and potentials, the studies were investigated with the collections of subjective assessments via user survey [7,11,12]. Moreover, studies that analyze usage log data were limited to simple log data, such as log-in or log-out, or stationary data [11,12,22]. The evaluations of the location of user access, or the mEMR usage according to time, were insufficient in determining the inherent value of the mobility [1,11,12,15,22].

Additionally, the analyses for the differences of mEMR usage among health care providers were insufficient, although there are a lot of documented differences for workflows by doctors and nurses [24-26]. Typically, doctors are full-time workers, with a few night shifts in their work schedule. Decision making and treatment plans for patients are determined mainly during the morning rounds [27-29]. However, the nurses work in three shifts that are carried out even in the middle of the night [30,31]. The differences in information needs, working hours, and workflow will be revealed as differences in usage patterns [26]. EMRs specialized in nursing are efficiently used by nurses with satisfaction [32]. Similarly, mEMRs require occupation-specific services. For more efficient use by each occupation in real-world practice, a detailed analysis of mEMR usage patterns and collection of related data are necessary.

In this study, we analyzed the usage pattern of health care providers and the wireless network access in the mobile environment based on mEMR data logs over a period of 4.5 years in an effort to overcome limitations of previous studies. Specifically, we investigated the peak time of usage and the differences and similarities in usage patterns between user types.

## Methods

### Study Design

To identify and verify the usage pattern of the mEMR according to time, user type, and wireless network, the mEMR usage log data were analyzed from March 2012 to August 2016. Usage log data were classified according to user occupations. Specifically, the analysis of the usage data determined the following: (1) the status of the mEMR usage and the distribution of users, (2) trends in the number of users and usage amount as a function of time (over a period of 4.5 years), (3) dissemination of 24-hour usage patterns (that exhibited differences based on yearly trends, trends based on user occupations, and usage patterns according to network access), and (4) trends in service usage based on user occupations. After analyzing the mEMR usage logs, the usage status of the system and the peak time usage between user occupations were described.

### Study Subjects and the mEMRs

**Figure 1:**
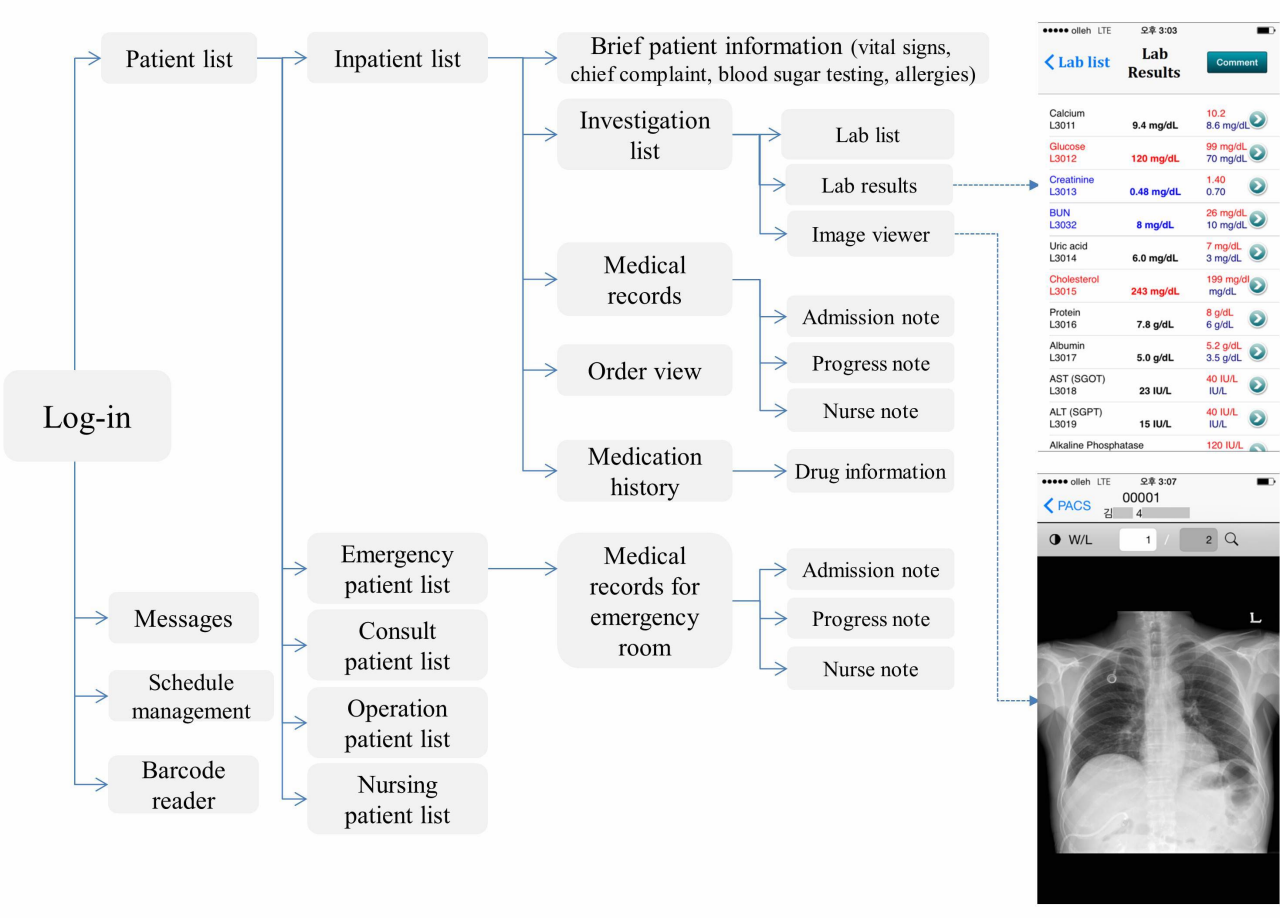
Function list and screen captures of the mobile Asan Medical Information System application. This application includes functions that allow viewing of all medical records, including medications, laboratory results, and images of radiologic studies.

This study was performed at the Asan Medical Center (AMC), the largest medical center in South Korea with more than 2700 beds, including 205 beds in intensive care units. The average count of daily outpatient visit was more than 11,600, and the average count of daily emergency room visit was more than 300 in 2016. The total number of employees in 2012 was 7408 (doctors: 1614, nurses: 3249), which increased to 7921 (doctors: 1676, nurses: 3605) in 2016. Since its establishment, the hospital information system, known as the Asan Medical Information System (AMIS), has been actively used [33]. The mobile version of the AMIS, the mobile AMIS (mAMIS), was launched in November 2010 (Figure 1) [10,16,22]. The functions and menus were selected and developed after gathering the opinions of health care workers based on surveys. The mAMIS version 2.0 was launched in March 2012 with extended functions, particularly for the usage of nurses. Additionally, three regular and three minor updates have been performed from the time of the launching to August 2016 [16,22]. It was initially based on iPhone operating system (iOS, Apple Inc), but the Android version was launched in 2013 [16].

Users can access patient information with the mAMIS in or out of the hospital based on implemented security and privacy systems. Using a certified user’s identification number and password, users could download the app from the app store of AMC via the hospital intranet (Wi-Fi network). Using JavaScript object notation, the app communicates with the hospital gateway server that controls direct access to the legacy database via device certification and encryption functions. Thereafter, the gateway server communicates with the legacy system (hospital information system) [8].

### Collection and Analysis of Usage Data

All system event logs were automatically stored in the mAMIS database server with the information of the user occupation, the event time, access page, and Internet protocol (IP) address (Wi-Fi information). To check the status of the system usage, the mAMIS version 2.0 log data were retrieved collectively from the server. No personal identifiers were gathered. The IP address was used to determine hospital network access. Only the mAMIS version 2.0 log data were used because of the difficulty of interpretation of log data acquired by different software versions.

We investigated the log data to identify trends regarding the actual usage of the mAMIS, overall usage patterns, usage characteristics of certain user occupation groups, and Wi-Fi network access patterns. To characterize the trend of the mAMIS usage, we analyzed the log data using linear regressions, and plotted the resulting trend lines in the corresponding figures. To determine the peak usage hour within a 24-h usage cycle, we investigated the time and usage amount of each local maximum point, that is, the point at which the value is greater than those of the adjacent points [34]. The differences between the groups were examined via a Student *t* test for categorical variables. All reported *P* values were two-sided, and *P* values less than .05 were considered significant. Data analyses were conducted with the R software version 3.3.1(The R Project for Statistical Computing).

This study was approved by the institutional review board of the hospital (IRB no. 2016-0287). The need for informed consent was waived by the ethics committee, as this study utilized routinely collected log data that were anonymously managed at all stages, including data cleaning and statistical analyses.

## Results

### User Characteristics

The mAMIS log data comprised 7,144,459 accumulated logs created by 3859 users between March 2012 and August 2016. Among the 3859 users, 2333 (60.46%) were nurses and 1102 (28.56%) were doctors. In 2015, the numbers of AMC doctors and nurses who used the mAMIS were 1882 and 3504, respectively, representing almost two-thirds of the health care workers (65.5% of the total number of doctors, and 66.6% of the total number of nurses). Other health care providers, such as health care assistants (n=194), pharmacists (n=68), and other staff members in management departments (n=162), accounted for the remaining users. Among the doctors, more than half of the users were trainees, that is residents and interns (58.90%, representing a ratio of 649/1102).

### Overall Usage Trend

**Figure 2:**
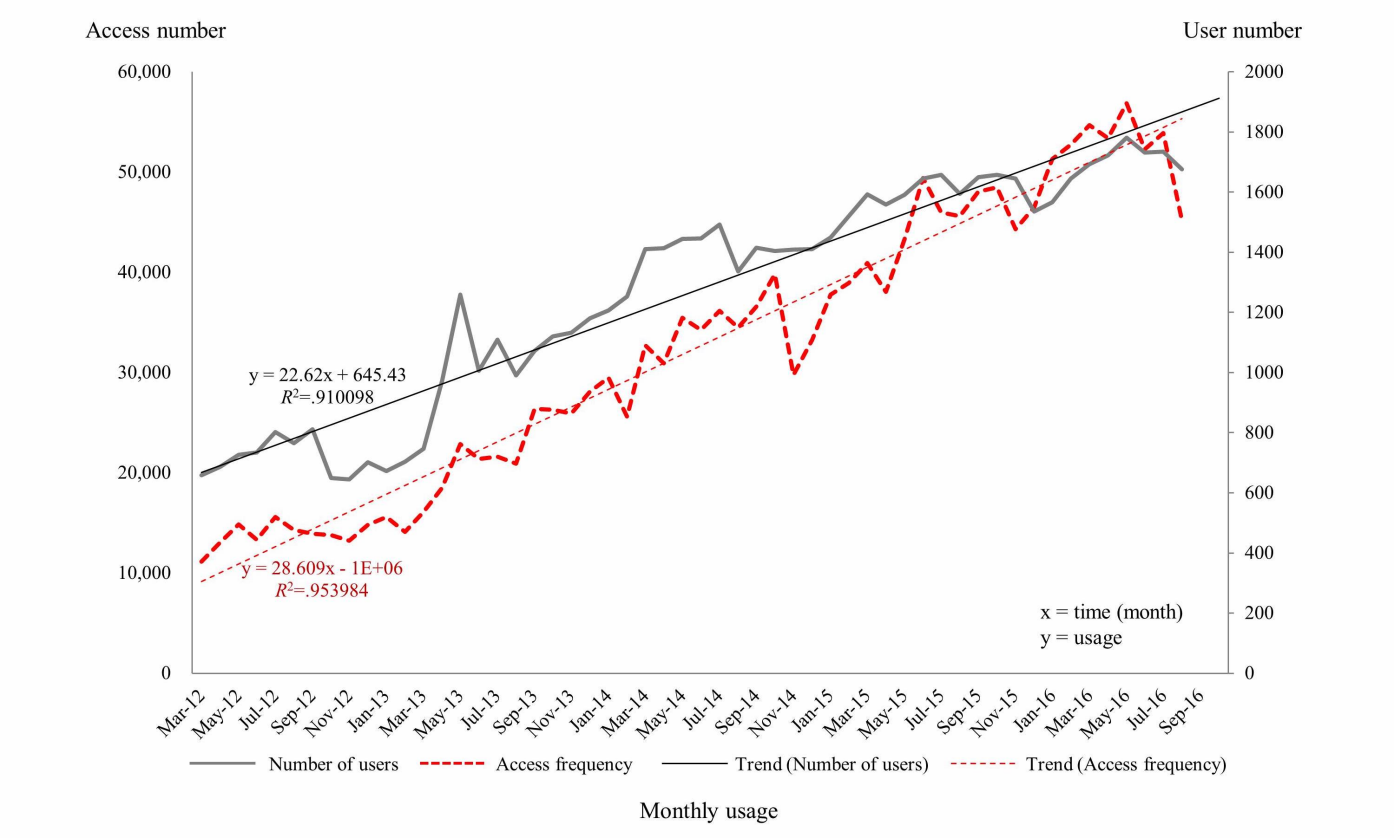
Monthly trends of the number of mobile Asan Medical Information System users and access frequencies from March 2012 to August 2016. The corresponding trend line and the correlation coefficient for usage per month present significant increases over time.

The access frequency and number of users increased throughout the period of data collection (Figure 2). To ascertain whether the access frequency and the number of users continuously increased, linear regressions were performed to model the relationship between the time and access frequency, and the number of users. The access frequency and the number of users exhibited abrupt and significant increases over time (estimated slopes=23.03 and 29.33 for the number of users and access frequency, respectively, *P*<.001 for both variables). The correlation coefficient for usage per month was high (*R*^2^>.9) in all cases.

### Hourly Usage Pattern According to User Occupation

Overall, the mAMIS was accessed, on average, 1044 times per day by 438 users. Of the 438 daily users, doctors accounted for 28.5% (125/438), and nurses accounted for 60.5% (265/438). When we examined the hourly usage according to user occupation, we found that on average, the nurses used the system 3 times more often than the doctors (60,481.9 vs 21,646.1) per day (Table 1).

**Figure 3:**
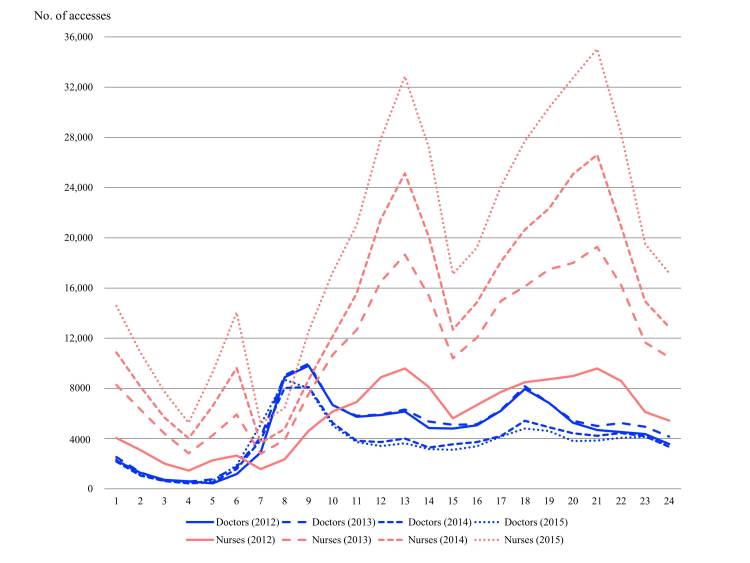
Annual trends in hourly usage of the mobile Asan Medical Information System. The access count for nurses increased by an annual average of 51.5%; however, for doctors, the access count decreased by an annual average of 7.7%.

The patterns of usage of the mAMIS between doctors and nurses were very different in terms of usage rates based on year and time (Figure 3). Initially, the use of mAMIS by nurses increased by an annual average of 51.5%, but the number of doctors who used it decreased by an annual average of 7.7%.

**Table 1:** Comparison of hourly wireless network access to the mobile Asan Medical Information System in according to user occupation.

Access count		Doctors	Nurses	Total
**Total access, n (%)**				
	Hospital Wi-Fi	144,303 (42.24)	197,286 (57.75)	341,589
	Nonhospital network	375,204 (23.02)	1,254,280 (76.97)	1,629,484
	All wireless networks	519,507 (26.35)	1,451,566 (73.64)	1,971,073
**Average access per hour (SD)**				
	Hospital Wi-Fi	6012.6 (4858.3)	8220.3 (3732.6)	14,232.9 (7161.2)
	Nonhospital network	15,633.5 (7350.4)	52,261.7 (26,075.2)	67,895.2 (30,143.7)
	All wireless networks	21,646.1 (11,660.8)	60,481.9 (29,431.8)	82,128.0 (35,393.1)
**Minimum access period^a^(times^b^)**				
	Hospital Wi-Fi	541 (4-5)	1946 (6-7)	3710 (3-4)
	Nonhospital network	2056 (3-4)	13,267(3-4)	15,323 (3-4)
	All wireless networks	2668 (3-4)	15,772 (6-7)	19,033 (3-4)
**Highest peak usage period^c^(times)**				
	Hospital Wi-Fi	19,095 (8-9)	15,159 (12-13)	26,284 (8-9)
	Nonhospital network	27,989 (7-8)	97,182 (20-21)	115,973 (20-21)
	All wireless networks	46,739 (8-9)	108,276 (20-21)	130,920 (20-21)
**Second peak usage period (times)**				
	Hospital Wi-Fi	11,124 (17-18)	12,309 (17-18)	23,541 (12-13)
	Nonhospital network	22,906 (17-18)	87,509 (12-13)	105,435 (12-13)
	All wireless networks	34,030 (17-18)	102,668(12-13)	128,976 (12-13)
**Third peak usage period (times)**				
	Hospital Wi-Fi	8382 (12-13)	11,094 (20-21)	23,433 (17-18)
	Nonhospital network	19,832 (21-22)	33,890 (5-6)	40,653 (5-6)
	All wireless networks	26,308 (12-13)	38,710 (5-6)	46,552 (5-6)

^a^Minimum duration (in hours) of user access during a 24-hour period.

^b^Time presentation follows a 24-hour notation.

^c^Maximum duration (in hours) of user access during a 24-hour period.

**Figure 4:**
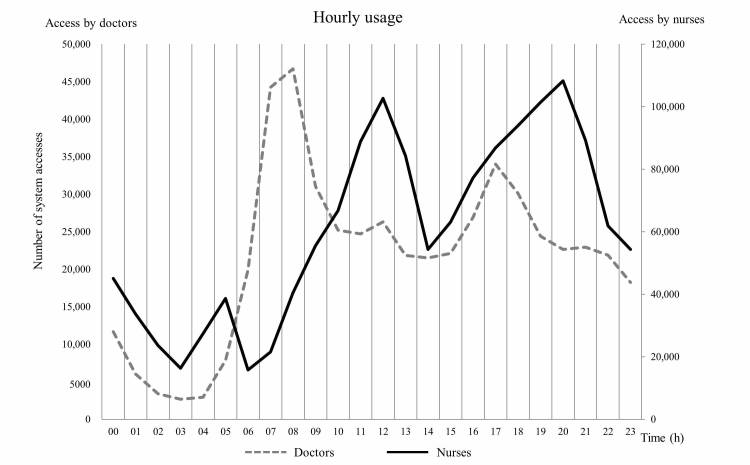
Hourly usage trends of mobile Asan Medical Information System according to user occupations.

Figure 4 indicates that the peak use time differs in the case of doctors and nurses. Specifically, we analyzed the usage rates according to the time of the day and found that the peak usage periods were different for each occupation (Table 1; Figure 4). The time periods in which definite peak usage was observed for doctors occurred from 08:00 to 09:00 and 17:00 to 18:00, with two minor peaks observed between 12:00 and 13:00 and 21:00 and 22:00. Conversely, peak usage for nurses was observed between 05:00 and 06:00, 12:00 and 13:00, and 20:00 and 21:00. The peak usage periods for doctors overlapped with times for ward rounds, whereas the peak usage periods for nurses occurred 1 to 2 hours before the handover time in a three-phase rotation system beginning at 07:00. Among the doctors, the usage associated with the highest peak (08:00-09:00) was 2.2 times higher than the average usage (46,739/21,646.1). Among the nurses, the usage associated with the highest peak (20:00-21:00) was 1.8 times higher than the average usage (108,276/60,481.9).

**Figure 5:**
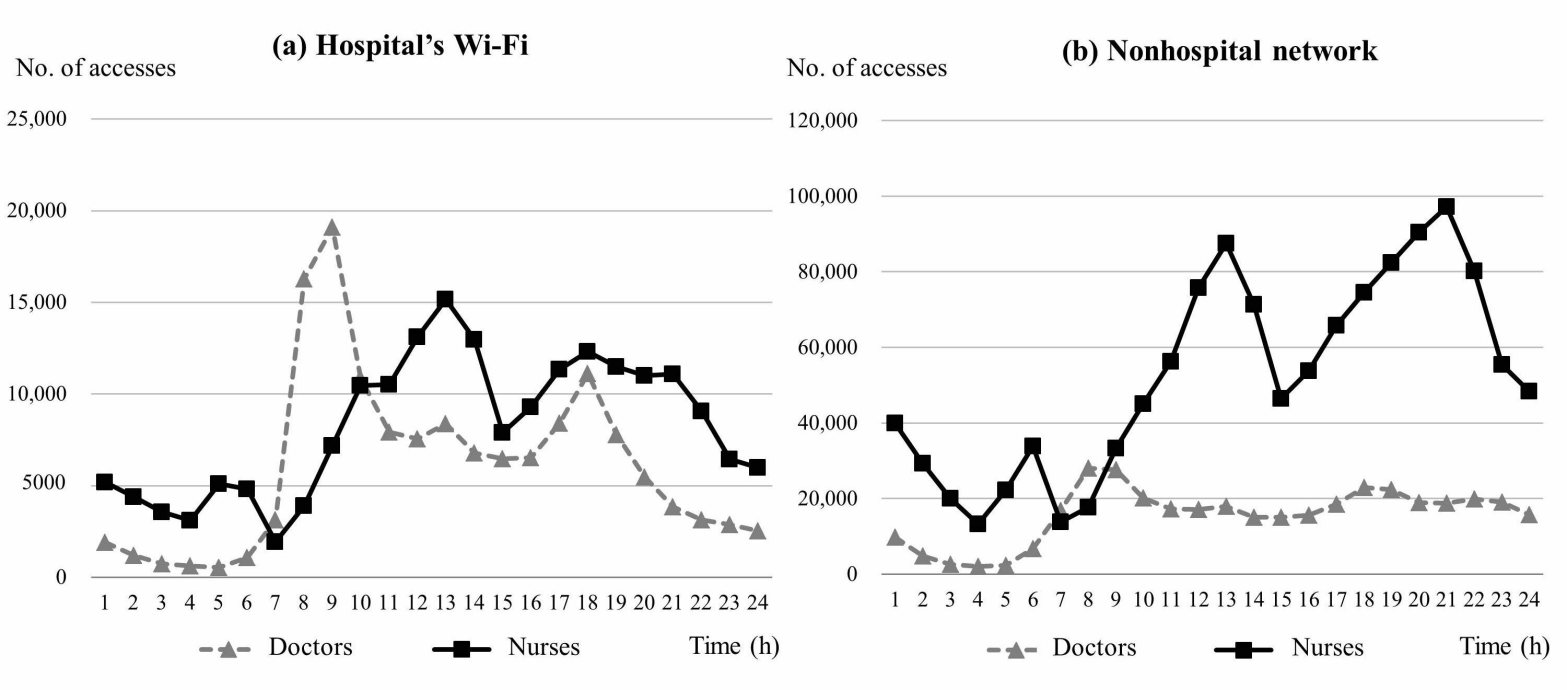
Network access frequency according to time and user’s occupations for (a) hospital’s Wi-Fi, and (b) a nonhospital network.

Throughout the data collection period, the mAMIS was accessed 1,971,073 times via a wireless network (Table 1). According to the network log data, more than 80% of accesses occurred via a nonhospital wireless network (1,629,484, 82.67%). Figure 5 illustrates the network access frequency of the hospital’s Wi-Fi and other nonhospital networks according to time and user occupation. On the basis of the hospital’s Wi-Fi use, there was no significant difference in access rates and averages between doctors and nurses (access rate: 42.2% vs 57.8%, average: 6012.6 vs 8220.3). Conversely, with nonhospital network use, nurses were found to access the system nearly 4 times more than doctors (access rate: 77.0% vs 23.0%, average: 52,261.7 vs 15,633.5).

### Service Menu Usage Pattern According to User

Table 2 provides a list of highly ranked mAMIS services and frequencies according to occupation. The highly ranked services list shows that the doctors and nurses utilize different services. For doctors, the “inpatient list” service was highest in frequency, followed by “lab results” and “lab list” services. Over 60% of system usage comprised these three services. For nurses, the “order view” service was highest in frequency, followed by “nurse note,” “nursing patient list,” and “EMR view” services. Approximately 90% of system usage by nurses comprised these four services.

**Table 2:** Ranking of mobile Asan Medical Information System services based on user occupations.

Function list	Nurse		Doctor	
	Rank	Access frequency, n (%)	Rank	Access frequency, n (%)
Order view	1	1,322,717 (23.90)	9	46,491 (2.90)
Nurse note	2	1,229,581 (22.22)	6	84,532 (5.24)
Nursing patient list	3	1,183,876 (21.39)	14	0 (0.00)
EMR^a^ view	4	1,096,258 (19.81)	4	134,721 (8.36)
Lab results	5	219,285 (3.96)	2	389,156 (24.14)
Lab list	6	142,787 (2.58)	3	201,535 (12.50)
EMR for ER^b^	7	87,077 (1.57)	10	27,670 (1.71)
Emergency patient list	8	85,549 (1.54)	8	48,705 (3.02)
Investigation list	9	53,235 (0.96)	5	128,507 (7.97)
Inpatient list	10	39,798 (0.71)	1	421,103 (26.13)
Operation patient list	11	28,859 (0.52)	12	23,976 (1.48)
PACS^c^ viewer	12	28,193 (0.50)	7	74,680 (4.63)
Drug information	13	15,376 (0.27)	13	3333 (0.20)
Consult patient list	14	433 (0.00)	11	27,026 (1.67)
Total		5,533,024		1,611,435

^a^EMR: electronic medical record.

^b^ER: emergency room.

^c^PACS: picture archiving and communication system.

## Discussion

### Principal Findings

According to the analyses, daily users increased from 200 in 2012 to 438 in 2016. The number of users and connections steadily increased over time. The steady use of approximately two-thirds of all health care providers over a 4-year period implies that the mAMIS had been well implemented and used reliably. Additionally, the differences in working hours and workflow between user occupations (doctors vs nurses) were reflected in the usage patterns. The doctors used the mAMIS more during the morning rounds, and the nurses used it more before the handover time. In both occupations, the users intensively used the mAMIS at the time when communication and information needs were high.

### Annual Usage Trends of the mAMIS

Compared with nurses where increase in usage was continuous, the usage by doctors decreased year by year. Although usage by nurses steadily increased at all time intervals, usage by doctors declined from 08:00 to 22:00, particularly between 2013 and 2014. The noted increase for nurses implies that the version with extended functions reflects user demands and leads to an increase in the actual usage. The first version of the mAMIS exhibited a higher proportion of doctor users than nurse users (doctors 66.0%; 416/630, nurses 31.0%; 195/630), and the percentage of users reversed in the early 2013. However, there was little difference in yearly usage by doctors from night time (10:00) to morning peak hours (08:00), which means that there were essential demands in the morning, regardless of the number of floating users.

The second version of the mAMIS was upgraded on the basis of user surveys. If we consider an mEMR based on the concept of the personal digital assistant, which was launched in 2004 and accessed only twice a day on average, the access rate of the mAMIS has markedly increased (2 vs 82,217), whereas the number of health care providers in AMC increased 1.6 times during the past 10 years (5092/3195) [22]. The improvement of function reflecting user feedback is essential for the hospital information system, which also applies to mEMRs [35]. Moreover, the number of doctor users is expected to increase through service improvements.

### Hourly Usage Pattern of the mAMIS

The peak usage periods of doctors overlapped with the starting times of morning and evening ward rounds (Figure 4). The highest peak occurred early in the morning when doctors evaluated the overnight events and the current status of patients before they began their rounds. The second peak occurred between 17:00 and 18:00, which was associated with most of the evening rounds. The two peak usage periods generally occurred when most of the doctors were actively moving through the hospital, and the access to patient information was needed to check the patient status. Timely usage of the mAMIS occurred during the peak hours, as the usage during the morning peak hour was more than twice as high as the average (46,739/21,646.1). This implies that the mAMIS has a unique value for doctors in assisting the preparation or execution of ward rounds.

The attendance of ward rounds necessitates the conduct of physical examinations, retrieval of patient information, and communication with other health care providers to make optimal decisions [27-29,36]. Because the ward round is such an active process, the most updated information should be exchanged, as doctors visit each patient [37]. Moreover, efficient information exchange and communication can lead to effective work and rational decision making [38,39]. Active users provided feedback via user surveys administered by the medical information office, indicating that they frequently used the system during rounds. However, it was not investigated whether the system was universally used during ward rounds.

The peak usage periods by nurse groups occurred between 05:00 and 06:00, 12:00 and 13:00, and 20:00 and 21:00. These time periods were 1 to 2 hours before handover times in a three-phase rotation system. The working patterns of the day-shift workers seemed to be reflected in the usage logs. The peak usage period and the use of a predominantly nonhospital network imply that users access the mAMIS while they are in transit to work. Additionally, the user survey revealed the mAMIS usage during commuting time. Correspondingly, the mAMIS possesses a unique role as a buffer between shifts, as the users prepare for the handover process during their spare time.

There was speculation that a flexible work time schedule can increase work efficiency; however, this cannot be applied to health care providers in a hospital [40,41]. An mEMR could compensate for the lack of flexibility, particularly for nurses who are obliged to adhere to a strict three-shift schedule. As a significant amount of information is exchanged within a short period of time during handover, checking information in advance could reduce the memory load. With the assistance of mEMRs, nurses can access the information of patients who will be under their care as they commute to work. This implies that nurses seemed to use the mAMIS as a tool for efficient workflow, particularly during the preparation for the handover. Moreover, as a successful handover influences patient safety, mEMR can improve patient safety, ensuring an efficient handover and reducing memory load [30,31,42]. Therefore, the development of specialized services that help nurses to precheck the tasks during the next shift could increase the usage by nurses.

Despite the extensive use of PC-based EMRs, there were several disadvantages [18-21,43]. PC-based systems are stationary, whereas health care providers are obliged to be mobile [19,43,44]. Furthermore, it can impede face-to-face communication [21]. For both doctors and nurses, the mAMIS was highly used in situations relevant to patient information access, and efficient communications were critical, such as ward rounds or handover. In addition, the peak usage time corresponded to the times when the users were actively moving. As the mAMIS was actively used during ward rounds or handover, mEMRs could fill in the gap between bedside and workstation, as well as promote work efficiency.

### Wireless Network Access of the mAMIS

Nurses accessed the mAMIS using a nonhospital wireless network manifested by the overwhelming usage majority. The nonhospital network access pattern of the nurses was comparable with the total wireless access pattern. It means that the system was used several times before or after regular business hours. As mentioned earlier, it is presumed that it was caused by usage during commuting time. Nurses accessed the system nearly 4 times more than doctors via a nonhospital network. Furthermore, more than 80% of accesses were via a nonhospital wireless network. This means that nurses did not use it much during their regular work.

Among the doctors, the amount of nonhospital network access began to increase compared with hospital Wi-Fi access, which was more prominent during the morning and evening peak usage periods. The hospital’s Wi-Fi access pattern for the doctors was comparable with their total wireless access pattern, although nonhospital network access was more common. The third usage peak of the hospital’s Wi-Fi that occurred during lunchtime implies the retrieval of information in a PC-free environment (Table 1). After the evening rounds, and up to midnight hours, negligible change in usage was observed via nonhospital network access (Figure 5). Nonhospital network access seemed to be used as a means of patient information access before and after working hours. This implies that because of the continuity of the patient care, doctors cannot be completely free of responsibility for their patients, even during off-duty time [42].

A low percentage of in-hospital usage means that the nurses did not sufficiently use the mAMIS during their work in the hospital. According to the studies showing that PC on wheels is efficient in rounds for nurses, nurses also have needs for mEMR in the hospital [45]. There is still plenty of room for improvement for in-hospital usage, such as new functions for nurse rounds. More efforts are needed to converge and reflect active user feedback, as the number of nurse users is growing.

The increased use of the nonhospital network by doctors may indicate the connections from outside, but it could be caused by troublesome hospital Wi-Fi. Nonhospital network access during regular work hours implies that doctors were reluctant to use the hospital’s Wi-Fi. Hence, the immediate access to patient information is the key value for the mAMIS, and hesitation for use because of network accessibility is a critical drawback. Increased nonhospital network access suggests the need to provide a more accessible Wi-Fi environment by adding Wi-Fi access points and implementing Wi-Fi interference solutions.

According to the majority of the mAMIS usage that occurred through the use of the nonhospital network, it is important to maintain a high level of security and to strengthen the information protection policies. Building stronger protection and security mechanisms will enable mEMRs to be used in a safer environment for both occupations [46-48]. Moreover, an improved network environment will enable the mEMRs to be used in a timely manner, reducing the working time. Therefore, regardless of whether the mAMIS is accessed inside or outside the hospital, security should be concerned when a nonhospital network is used [15,46-48].

### Usage Pattern According to Service Menus

The menus accessed by doctors and nurses were significantly different. Among the doctors, the most frequently accessed service was the “inpatient list,” followed by “lab results” (Table 1). These results showed that checking real-time lab results is important, as it may require immediate action [37]. Moreover, this implies that easier access to the lab results of patients of special concern, such as patients in critical conditions, may facilitate a more efficient workflow [11]. With the exceptions of “inpatient list” and “lab list” functions, a gradual decline in the usage amount was observed. Additionally, the increased usages of “EMR views” and “nurse notes” among the doctors suggest the existence of the need to identify overnight events for the patients in their care, before the onset of routine work. Similar findings were observed in the case of the nurse group. However, because there was no information on time-service usage, it was not possible to confirm this assumption. Considering the increased usage of certain services during the peak periods, it would be helpful to add a quick menu option that is customized for rounds or shortcuts to frequently used menus.

Conversely, the usage logs from the nurse group showed increased utilization over specific services (order list, nurse notes, nursing patient list, and EMR views). The usage log count of the fifth most highly used service among the nurses, namely, “lab results,” equaled one-fifth of the fourth most highly used service (219,285/1,096,258), that is “EMR views.” Due to the nature of their work, “order view” seems to be the most frequently accessed menu option among the nurses. Among the top five menu options accessed by the doctors, “EMR views” was the only menu option that was also included in the “top four” menu options accessed by the nurses. This finding confirms the necessity for a more simplified but specialized service menu for nurses. Furthermore, it is necessary to actively develop useful menus for more practical use, considering that the use of menus among the nurses was limited, and that most of the services were accessed via a nonhospital network. Considering that there is increased use during the handover preparation, it would be helpful to develop a menu customized for efficient handover.

The mAMIS system should be used more frequently in clinical practice or point of care as was originally intended [22]. Diverse functions for use at bedside could be implemented, such as structured data entries for doctors, medication administration records for nurses, and barcode applications for patient identification. A user-customized service, or menu arrangement, could also facilitate task-oriented usages.

### Limitations

As this study was performed with usage data from a single medical center, there are several limitations to extrapolating these results to all medical centers. First, most functions of the mAMIS are for reading or viewing, and not for writing or data entries. Analysis of mEMRs that contain balanced writing and viewing functions could be considerably different. Second, although access logs of menus were displayed, detailed usage logs were not analyzed because the user-specific access data were insufficient. If we could obtain such data, we would be able to investigate which users most often accessed the mAMIS, which menu options were most accessed during peak usage times, and so on. Third, active user surveys or interviews would be helpful to demonstrate our assumption; however, the pursuit of these studies was outside the scope of our study. The results of surveys on user perception and usage patterns, in addition to coordinating these results with actual log data, could provide definitive evidence of the value of the mEMRs.

### Conclusions

The usage data of the mAMIS proved valuable for communication between health care providers and continuity of patient care. Although the usage pattern considerably varied between doctors and nurses, the mAMIS was accessed by users in circumstances of active movement. To improve the usability of the mAMIS, more user-centered service developments are required in addition to improvements to the user environment, such as a more accessible Wi-Fi network and increased security. Additional studies based on real-world data and clinical preferences should be considered to evaluate user satisfaction and the clinical implications of the mEMR.

## Abbreviations

EMRs: electronic medical records
IP: Internet protocol
mEMR: mobile electronic medical record
mAMIS: mobile Asan Medical Information System
PC: personal computer
